# Expression of fibroblast growth factor receptor family members is associated with prognosis in early stage cervical cancer patients

**DOI:** 10.1186/s12967-016-0874-0

**Published:** 2016-05-06

**Authors:** Chel Hun Choi, Joon-Yong Chung, Jae-Hoon Kim, Byoung-Gie Kim, Stephen M. Hewitt

**Affiliations:** Experimental Pathology Laboratory, Laboratory of Pathology, National Cancer Institute, Center for Cancer Research, National Institutes of Health, MSC 1500, Bethesda, MD 20892 USA; Department of Obstetrics and Gynecology, Gangnam Severance Hospital, Yonsei University College of Medicine, Seoul, 135-720 Korea; Department of Obstetrics and Gynecology, Samsung Medical Center, Sungkyunkwan University School of Medicine, 50 Irwon-dong, Gangnam-gu, Seoul, 135-710 Republic of Korea

**Keywords:** Fibroblast growth factor receptor, Image analysis, Immunohistochemistry, Prognosis, Survival analysis, Uterine cervical neoplasm

## Abstract

**Background:**

The oncogenic role of the fibroblast growth factor receptor (FGFR) has been recognized in a number of different cancer types. However, the prognostic significance of FGFRs has not been elucidated yet in cervical cancer. In the present study, we investigate the expression of FGFRs and their prognostic value in cervical cancer patients.

**Methods:**

FGFR1, FGFR2, FGFR3, and FGFR4 expression was determined by immunohistochemistry in conjunction with quantitative digital image analysis of 336 formalin-fixed, paraffin-embedded cervical cancer tissues and 61 normal cervical tissues, as well as NCI60 cell microarray. Subsequently, the association between clinicopathological characteristics and patient survival was assessed.

**Results:**

FGFRs proteins were differentially expressed in the NCI60 cell line panel and showed considerable correlation between protein and mRNA expression. The expression of FGFR1, FGFR2, and FGFR4 were higher in cancer tissues than in normal tissues, whereas the expression of FGFR3 was higher in normal tissues. FGFR1 was highly expressed in adeno-/adenosquamous carcinoma (*P* = 0.020), while FGFR2, FGFR3, and FGFR4 expression were more prominent in squamous cell carcinoma (*P* < 0.001, *P* < 0.001, and *P* = 0.020, respectively). FGFR2 expression was significantly higher in small sized tumors (*P* = 0.020). Additionally, high FGFR2 and FGFR4 were correlated with negative lymph node metastasis (*P* = 0.048 and *P* = 0.040, respectively). FGFR1, FGFR2, and FGFR3 were highly expressed in tumors without parametrial involvement (*P* = 0.030, *P* = 0.005, and *P* = 0.010, respectively). In survival analysis, high expressions of FGFR2, FGFR3, and FGFR4 was associated with longer disease-free survival (*P* = 0.006, *P* = 0.035, *P* = 0.001, respectively) and overall survival (*P* = 0.003, *P* = 0.002, *P* = 0.003, respectively). Notably, the co-expression of all three FGFRs was significantly associated with favorable disease-free survival (*P* < 0.001) and overall survival (*P* < 0.001), compared to the negative expressions of the three FGFRs. The prognostic significance persisted in the cox regression analysis.

**Conclusions:**

The frequent expression of members of the FGFR family in cervical cancer suggests they may have prognostic and therapeutic relevance.

**Electronic supplementary material:**

The online version of this article (doi:10.1186/s12967-016-0874-0) contains supplementary material, which is available to authorized users.

## Background

Cervical cancer is the third most common type of cancer among women worldwide, and is the most prevalent female malignancy in many developing countries [1, 2]. Although preventive vaccination and screening are good in prevention, invasive cancer continues to occur, even among women who have access to cancer screening, and the prognosis remains poor in patients with a bulky tumor or adenocarcinoma histology [3–5]. Clinical factors such as International Federation of Gynecology and Obstetrics (FIGO) stage, lymph node metastasis, and tumor size may serve as prognostic markers, however they are insufficient in accurately predicting recurrence and survival. Thus, biomarkers, including molecular markers, are needed. Patient care would be considerably improved if tumor behavior could be reliably prognosticated at the time of initial diagnosis.

The mammalian fibroblast growth factor (FGF) family comprises 18 ligands that exert their actions through four transmembrane tyrosine kinase receptors; fibroblast growth factor receptor (FGFR) 1, FGFR2, FGFR3, and FGFR4. The FGF/FGFR system is responsible for multiple cellular functions, including proliferation, differentiation, survival, and motility [6–9]. Considering these functions, it is no surprise they are susceptible to aberration in cancer cells [10]. Several types of genetic alterations have been recognized in cancers, including gene amplifications, activating mutations, chromosomal translocations, and aberrant splicing at the posttranscriptional level [10]. For instance, FGFR1 mutations and amplifications have been implicated in prostate [11], breast [12] and lung cancers [13], whereas aberrant FGFR2 expression has been observed in endometrial [14], breast [15], and gastric cancer [16]. Furthermore, activating mutations and overexpression of FGFR3 have been reported in bladder cancers [17] and multiple myeloma [18]. We have identified mutation in FGFR4, which acts as an oncogene, and suggested therapeutic targeting of FGFR4 in rhabdomyosarcomas [19]. In addition, we recently examined a prevalent occupational exposure susceptibility variants associated with increased bladder cancer risk, and observed an additive interaction for rs798766 (TMEM129-TACC3-FGFR3) with the interaction more apparent in patients with tumors positive for FGFR3 expression [20]. In cervical cancer, recent studies have shown the possible involvement of aberrant FGFR signaling with human papillomavirus (HPV) 16 E5 expression [21]. Notably, FGFR2 expression has been reported to be associated with cell growth and progression of cervical dysplasia [22, 23].

Recently, alterations in the FGFR signaling pathway have gained the spotlight due to their high incidence rate and therapeutic potential in malignancies [24]. Nevertheless, protein expression of the FGFR system and its prognostic significance in cervical cancer has not yet been elucidated. Thus, the aim of this study is to investigate the clinical significance of FGFR1, FGFR2, FGFR3 and FGFR4 expression in a well-defined cohort of cervical cancers, using immunohistochemistry and quantitative digital image analysis.

## Methods

### Patients and tumor samples

In the present study, we retrieved a total of 336 early stage cervical cancer patients treated in the Department of Gynecologic Oncology, Samsung Medical Center, Sungkyunkwan University School of Medicine between 2002 and 2009. Patients with rare histology such as sarcoma, malignant melanoma, and neuroendocrine carcinoma, and patients with limited availability of tissue block specimens were excluded from the tissue microarray (TMA) construction. 61 non-matched, non-adjacent normal epithelial tissues were used for the control. Tissue samples were collected from patients who had signed an informed consent form, which was approved by the Institutional Review Board at the Samsung Medical Center, Seoul, Korea (2009-09-002-002 and 2015-07-122). Some of the paraffin blocks were provided by the Korea Gynecologic Cancer Bank through Bio & Medical Technology Development Program of the Ministry of Education, Science and Technology, Korea (NRF-2012M3A9B8021800). This study was additionally approved by the Office of Human Subjects Research at the National Institute of Health.

All patients were treated primarily with radical hysterectomy with or without pelvic/para-aortic lymph node dissection. Patients received adjuvant radiotherapy with or without concurrent chemotherapy if they had risk factors. Following treatment, patients had follow-up examinations every 3 months for the first 2 years, every 6 months for the next 3 years, and once annually every year thereafter. Disease-free survival (DFS) was assessed from the date of surgery to the date of recurrence or the date of the last follow-up visit. Overall survival (OS) was measured from the date of surgery to the time of death, or for the living patients, to the date of last contact.

### Tissue microarray construction and immunohistochemistry

Tissue microarrays (TMAs) were constructed from tissue blocks used for routine pathological evaluation. In each case, areas with the most representative histology were selected, and cylindrical tissue samples (0.6 mm) were cored from the donor block and extruded into the recipient array. We included three cores from separate tissue blocks of each patient to reduce sampling bias.

NCI60 cell microarray was constructed as previously described [25]. In brief, NCI60 cells were collected and then fixed in 70 % ethanol. After fixation, cells were pelleted and added 3 % low-melt agarose (SeaPlaque, Cambrex, Rockland, ME). The cells were immediately evenly dispersed in the warm by vortexing, and placed on ice for 2 min to form agar plugs. The agarose plug was transferred into a histology cassette (Tissue-TekTM, Sakura Finetek, Torrance, CA) and then processed in Tissue-Tek VIP processor (Sakura) and subsequently paraffin-embedded to form a donor block. Finally, the NCI60 cell microarray was constructed using 1.0 mm needle.

Immunohistochemical staining of FGFR1, FGFR2, FGFR3 and FGFR4 was performed on 4-μm sections of the TMA and NCI60 cell microarray, as described previously [26]. In brief, following deparaffinization and dehydration, heat-induced antigen retrieval was performed for 20 min in an antigen retrieval buffer of pH 9.0 (for FGFR3 and FGFR4) or pH 6.0 (for FGFR1 and FGFR2) (Dako, Carpinteria, CA) using a steam pressure cooker (Pascal, Dako). The endogenous peroxidase activity was blocked with 3 % H_2_O_2_ for 10 min. For FGFR1 and FGFR2, sections were incubated with protein block (Dako) for further 15 min. The sections were subsequently incubated with primary antibodies, a detailed list of which, along with dilutions, is provided in Additional file 1: Table S1. The antigen–antibody reaction was detected with Dako EnVision+Dual Link System-HRP (Dako) and DAB+ (3, 3′-Diaminobenzidine; Dako). In the negative control sections, the primary antibody was omitted. Positive controls included umbilical cord, stomach carcinoma, skin tissue, and prostate cancer for FGFR1, FGFR2, FGFR3, and FGFR4 antibodies, respectively. Tissue sections were lightly counterstained with hematoxylin and examined by light microscopy.

### Quantitative evaluation of immunostaining

The evaluation of immunostaining was carried out using computer-assisted image analyzing software version 4.5.1.324 (Visiopharm, Hoersholm, Denmark,). Immunohistochemically stained slides were scanned using a NanoZoomer 2.0 HT (Hamamatsu Photonics, Hamamatsu City, Japan) at 20× objective magnification (0.5-μm resolution), and captured digital images were then imported into the Visiopharm software. Each core was imported separately using the TMA workflow of the program.

Digital images consist of pixels, each of which is defined by a position and a value of intensity [27]. After training the system by digitally “painting” examples of the nucleus in the image, tumor nuclei were defined (Additional file 2: Figure S1A–B). Cytoplasm is further defined by outlining the defined nucleus (Additional file 2: Figure S1C). The mean intensity of DAB for each defined image is used for quantification of the expressions (Additional file 2: Figure S1D). The intensity of staining was categorized as 0, 1+, 2+, and 3+ according to the distribution pattern across cores. The final score was calculated by multiplying the intensity and percentage of staining resulting in a final histoscore of 0–300 [28].

### Microarray gene expression profiling

To examine the prognostic significance of mRNA expression, microarray data were analyzed as described previously [29]. In brief, RNA was extracted from formalin-fixed, paraffin-embedded tissue sections with removal of nontumor elements. RNA was extracted using the High Pure RNA Paraffin kit (Roche Diagnostic, Mannheim, Germany) and subsequently, the whole genome cDNA mediated annealing selection and ligation (WG-DASL) assay was performed following the manufacturer’s instructions (Illumina, San Diego, CA). With the exclusion of inadequate samples, a total of 300 patient samples were evaluable and the expression data was deposited in the Gene Expression Omnibus (GEO) database (http://www.ncbi.nlm.nih.gov/geo/query/acc.cgi?acc= GSE44001).

### In-silico analysis for TCGA cervix

To evaluate the prognostic significance of *FGFR* mRNA expression, data from The Cancer Genome Atlas (TCGA) Research Network were also analyzed (http://cancergenome.nih.gov/). Pan-cancer normalized form of RNA-seq data of cervical cancers, which had been obtained using a Illumina HiSeq (Illumina, San Diego, CA, USA), were downloaded (version: 2015-02-24). For survival analysis, the mRNA expression value was dichotomized according to quartile values (lower than 25 percentile vs. higher than 75 percentile).

### Statistical analysis

Statistical analysis was performed using the R software version 3.1.2. The expression level of the proteins according to the clinicopathological characteristics were analyzed using a Student’s t test. Analysis of the Spearman rho coefficient was used to assess associations and correlations between parameters. For survival analysis, expression values were dichotomized (positive vs. negative) with the cut-off values showing the most discriminative power in the univariate cox model for disease-free survival (Additional file 2: Figure S1E) (R package: survMisc). Survival distributions were estimated using the Kaplan–Meier method, and the relationship between survival and each parameter was analyzed with the log-rank test. A Cox proportional hazards model was created to identify independent predictors of survival. Statistical significance was considered to be present at values of *P* < 0.05.

## Results

### Clinicopathological characteristics of patients

Clinicopathological characteristics of 336 patients are summarized in Table 1. Patients with IB2 or IIB who were treated primarily with radical surgery were included in the present study. One hundred and sixty patients (47.6 %) were treated with adjuvant radiation, with or without concurrent chemotherapy following radical surgery.

**Table 1:** Clinicopathological characteristics of the 336 cervical cancer patients

	Frequency	%
Age		
Mean ± SD	48.9 ± 11.2	
FIGO stage		
IB1/IIA	291	86.6
IB2/IIB	45	13.4
Cell type		
SCC	256	76.2
AD/ASC	80	23.8
Tumor size		
≤4 cm	256	76.2
>4 cm	80	23.8
SCC Ag level (ng/ml)		
Median (range)	1.2 (0.1–65.1)	
LVSI		
Negative	202	60.1
Positive	134	39.9
Depth of invasion		
<50 %	108	32.1
>50 %	228	67.9
LN metastasis		
Negative	256	76.2
Positive	80	23.8
PM involvement		
Negative	305	90.8
Positive	31	9.2
Resection margin		
Negative	323	96.1
Positive	13	3.9
Primary treatment		
OP only	171	50.9
OP + RT	70	20.8
OP + CCRT	90	26.8
Neoadjuvant	5	1.5

*FIGO* International Federation of Gynecology and Obstetrics; *SCC* squamous cell carcinoma; *Ag* antigen; *AD* adenocarcinoma; *ASC* adenosquamous cell carcinoma; *LVSI* lymphovascular space invasion; *LN* lymph node; *PM* parametrium; *OP* operation; *RT* radiotherapy; *CCRT* concurrent chemoradiotherapy

### FGFRs expression in the NCI60 cell lines

The NCI60 cell lines are widely used cancer biology and drug discovery. In addition, the NCI60 cell microarray is a useful platform for antibody validation because immunohistochemistry (IHC) data of NCI60 cell microarray are used to predict antibody titer for IHC on multi-tumor TMA [25]. Thus, we performed IHC on NCI60 cell microarray to assist in assessment of antibody specificity.

**Fig. 1:**
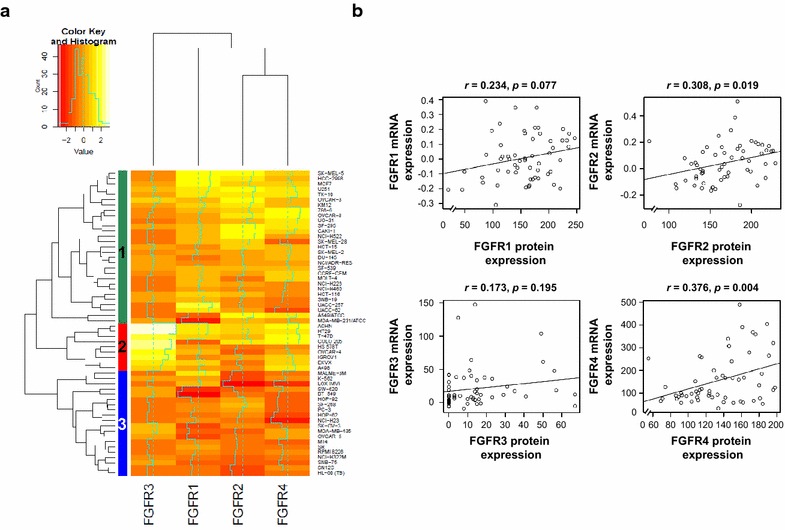
FGFRs expression in the NCI60 cell lines. **a** Hierarchical clustering analysis for immunohistochemical expression of FGFR1, FGFR2, FGFR3, and FGFR4. Three categories were defined. Category 1 (*n* = 29) consists relatively of low FGFR1, FGFR2, and FGFR4 expression. In contrast, category 3 (*n* = 20) consists exclusively of high FGFR1, FGFR2, and FGFR4 expression. Category 2 (*n* = 9) consists exclusively of low FGFR3 expression. **b** Correlation between FGFRs mRNA and protein expression. mRNA expression level was measured by microarray gene expression profiling, whereas the protein expression was assessed by immunohistochemistry. FGFR2 and FGFR4 protein expression was correlated with mRNA expression (*P* = 0.019 and *P* = 0.004, respectively)

A total of 58 cell lines were analyzed by hierarchical clustering with the continuous histoscore. As shown in Fig. 1, three categories were defined. Category 1 (*n* = 29) consists relatively of low FGFR1, FGFR2, and FGFR4 expression. In contrast, category 3 (*n* = 20) consists exclusively of high FGFR1, FGFR2, and FGFR4 expression. Furthermore, category 2 (*n* = 9) consists exclusively of low FGFR3 expression. FGFR2 and FGFR4 protein expression was correlated with mRNA expression (*r* = 0.308; *P* = 0.019 and *r* = 0.413; *P* = 0.004, respectively) (Fig. 1b). Although FGFR1 and FGFR3 did not meet statistical significance there is a positive correlation trend. These data suggest that FGFRs proteins were differentially expressed in the NCI60 cell lines and antibodies of FGFR isoforms had specificity.

### FGFR expression and its associations with clinicopathological features

**Fig. 2:**
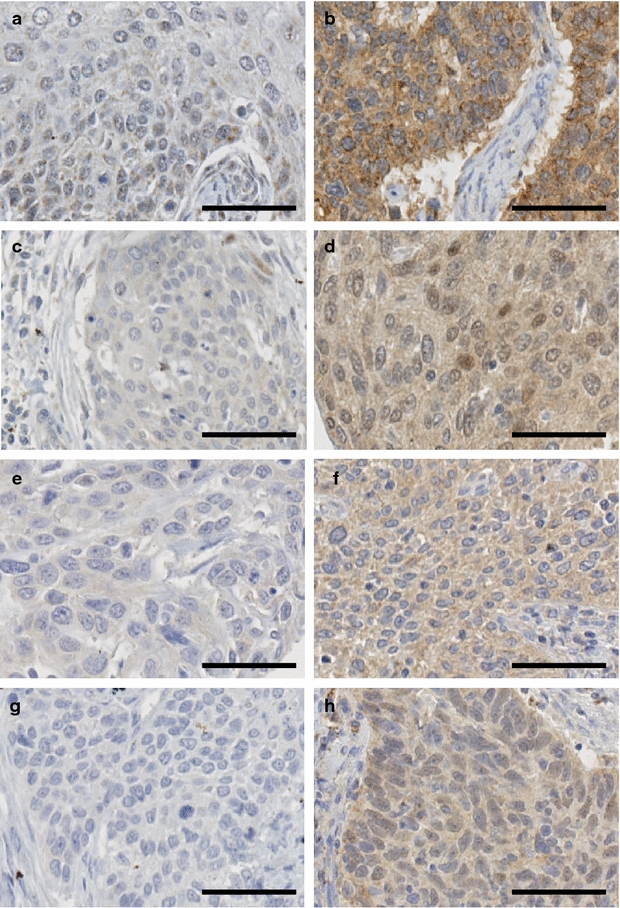
FGFR1, FGFR2, FGFR3, and FGFR4 expression in formalin-fixed, paraffin-embedded cervical cancer tissues. Representative immunohistochemical images of FGFR1 negative (**a**) and positive (**b**), FGFR2 negative (**c**) and positive (**d**), FGFR3 negative (**e**) and positive (**f**), FGFR4 negative (**g**) and positive (**h**) expression. The *Scale bar* represents 50 μm

FGFR1, FGFR2, FGFR3 and FGFR4 expression is mainly observed in the cytoplasm [30–32], and representative examples of positive and negative staining are shown in Fig. 2. Determination of the cut-offs for expression of FGFRs was also referenced to overall survival. The *P* value of the predicted log-rank test of Kaplan–Meier results was plotted against the normalized staining values (Additional file 2: Figure S2) and supports the determination of cut-offs for FGFR2, FGFR3, and FGFR4 based on the histogram. FGFR1 expression lacked as clear a relationship between expression and outcome by this analysis. Among the 336 tumors investigated, the number of tumors exhibiting high FGFR expression was 88 (26.2 %, histoscore >122) for FGFR1, 167 (49.7 %, histoscore >58) for FGFR2, 211 (62.8 %, histoscore >57) for FGFR3 and 241 (71.7 %, histoscore >79) for FGFR4. When compared with normal tissues, the expression of FGFR1, FGFR2, and FGFR4 were higher and the expression of FGFR3 was lower in cancer tissues (Table 2). The expression of FGFR was cell type associated. FGFR1 was more highly expressed in adeno-/adenosquamous carcinoma, while FGFR2, FGFR3, and FGFR4 expression was more prominent in squamous cell carcinoma (*P* = 0.020, *P* < 0.001, *P* < 0.001, and *P* = 0.020, respectively) (Table 2). These results suggest that each FGFR potentially has a different role according to cell type in cervical cancers.

**Table 2:** Correlation between FGFR expression and clinicopathological characteristics of cervical cancer

	FGFR1	FGFR2	FGFR3	FGFR4
	Mean histoscore [95 % CI]	Mean histoscore [95 % CI]	Mean histoscore [95 % CI]	Mean histoscore [95 % CI]
Normal vs. cancer				
Normal	35 [27–42]	32 [23–40]	106 [89–124]	68 [57–80]
Cancer	84 [77–91]	70 [64–77]	82 [76–87]	129 [122–136]
*P* value	<0.001	<0.001	0.02	<0.001
Stage				
IB1/IIA	82 [75–90]	72 [64–79]	83 [76–89]	130 [121–138]
IB2/IIB	89 [70–107]	66 [48–83]	79 [65–93]	117 [97–136]
*P* value	0.520	0.520	0.630	0.220
Cell type				
SCC	78 [71–86]	77 [69–85]	89 [82–96]	133 [124–141]
AD/ASC	99 [83–115]	51 [40–62]	59 [48–71]	113 [98–128]
*P* value	0.020	<0.001	<0.001	0.020
Tumor size				
≤4 cm	85 [77–93]	74 [667–83]	83 [76–90]	131 [122–139]
>4 cm	79 [64–91]	59 [47–70]	79 [68–89]	120 [105–134]
* P* value	0.330	0.020	0.500	0.210
LVSI				
Negative	84 [75–93]	73 [64–82]	84 [76–93]	137 [127–147]
Positive	82 [71–93]	67 [57–77]	78 [70–87]	114 [104–126]
* P* value	0.770	0.390	0.330	0.004
Depth of invasion				
<50 %	88 [75–101]	73 [60–86]	85 [74–97]	132 [118–146]
>50 %	81 [73–90]	70 [62–78]	80 [73–87]	126 [117–135]
*P* value	0.420	0.670	0.490	0.470
LN metastasis				
Negative	83 [75–91]	74 [66–82]	84 [77–91]	132 [124–141]
Positive	84 [70–98]	60 [48–72]	75 [65–83]	114 [98–129]
*P* value	0.920	0.048	0.160	0.040
PM involvement				
Negative	85 [78–93]	73 [66–80]	83 [77–90]	129 [121–137]
Positive	65 [48–82]	49 [34–64]	64 [50–78]	115 [91–139]
*P* value	0.030	0.005	0.010	0.250
Resection margin				
Negative	83 [76–90]	71 [64–78]	82 [76–88]	128 [121–136]
Positive	88 [50–127]	70 [42–99]	80 [52–108]	118 [75–161]
*P* value	0.780	0.970	0.890	0.620
Primary treatment				
OP only	84 [74–94]	81 [70–91]	91 [81–100]	136 [125–147]
OP + RT	81 [66–96]	67 [53–81]	73 [62–85]	127 [111–143]
OP + CCRT	83 [70–96]	54 [45–64]	71 [62–81]	115 [100–129]
Neoadjuvant	86 [2–171]	96 [17–175]	96 [20–173]	119 [38–199]
*P* value	0.990	0.051	0.120	0.180

*FIGO* International Federation of Gynecology and Obstetrics; *SCC* squamous cell carcinoma; *Ag* antigen; *AD* adenocarcinoma; *ASC* adenosquamous cell carcinoma; *LVSI* lymphovascular space invasion; *LN* lymph node; *PM* parametrium; *OP* operation; *RT* radiotherapy; *CCRT* concurrent chemoradiotherapy

In addition, the high expression of FGFR1, FGFR2, FGFR3 were negatively correlated with the parametrial involvement (*P* = 0.030, *P* = 0.005, and *P* = 0.010, respectively). Furthermore, FGFR2 expression was down-regulated in large-sized (*P* = 0.020) and lymph node metastatic (*P* = 0.048) tumors. FGFR4 expression was also down-regulated in lymphovascular space invasive (*P* = 0.004) and lymph node metastatic (*P* = 0.040) tumors. These results indicate that FGFR expression is associated with less aggressive phenotypes in cervical cancers.

**Fig. 3:**
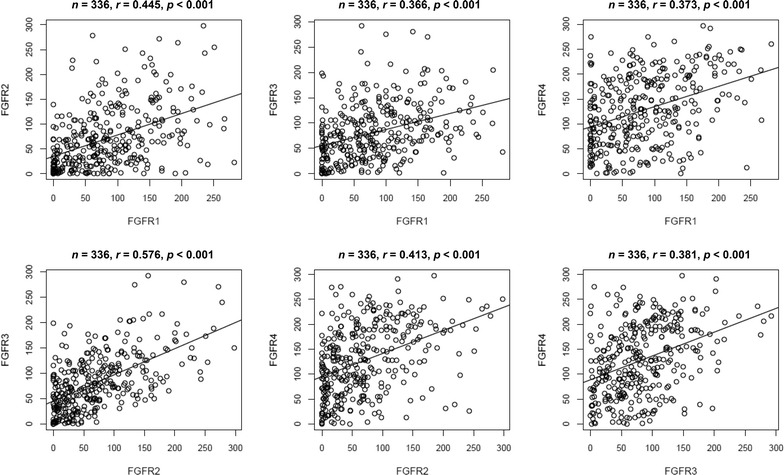
Relationship among FGFR1, FGFR2, FGFR3, and FGFR4 expression in cervical cancer tissues. There is a significant positive correlation between each FGFR expression (*P* < 0.001)

In order to find the clustering of samples according to FGFR expression, a total of 336 cervical cancer cases were analyzed by hierarchical clustering with the continuous histoscore. As shown in Additional file 2: Figure S3, two categories were defined. Category 1 (*n* = 116) consists exclusively of high FGFR2 and FGFR3 expression. In contrast, category 2 (*n* = 220) consists exclusively of low FGFR1, FGFR2, and FGFR3 expression. Furthermore, there are significant differences for cell type, tumor size, lymph node metastasis and primary treatment between category 1 and category 2 (Additional file 1: Table S2). Notably, category 2 associated with advanced tumor phenotypes and had poor prognosis. Subsequently, we examined the correlation among FGFR1-4 expression. FGFR1 expression was positively correlated with FGFR2, FGFR3, or FGFR4 expression (*r* = 0.445, *r* = 0.366 and *r* = 0.373, respectively; all *P* < 0.001). Furthermore, FGFR2 expression was positively correlated with FGFR3, or FGFR4 expression (*r* = 0.576, and *r* = 0.413, respectively; all *P* < 0.001). There was also a significant correlation between FGFR3 and FGFR4 expression (*R* = 0.381, *P* < 0.001) (Fig. 3).

### High expression of FGFR2, FGFR3, and FGFR4 predict longer survival

**Fig. 4:**
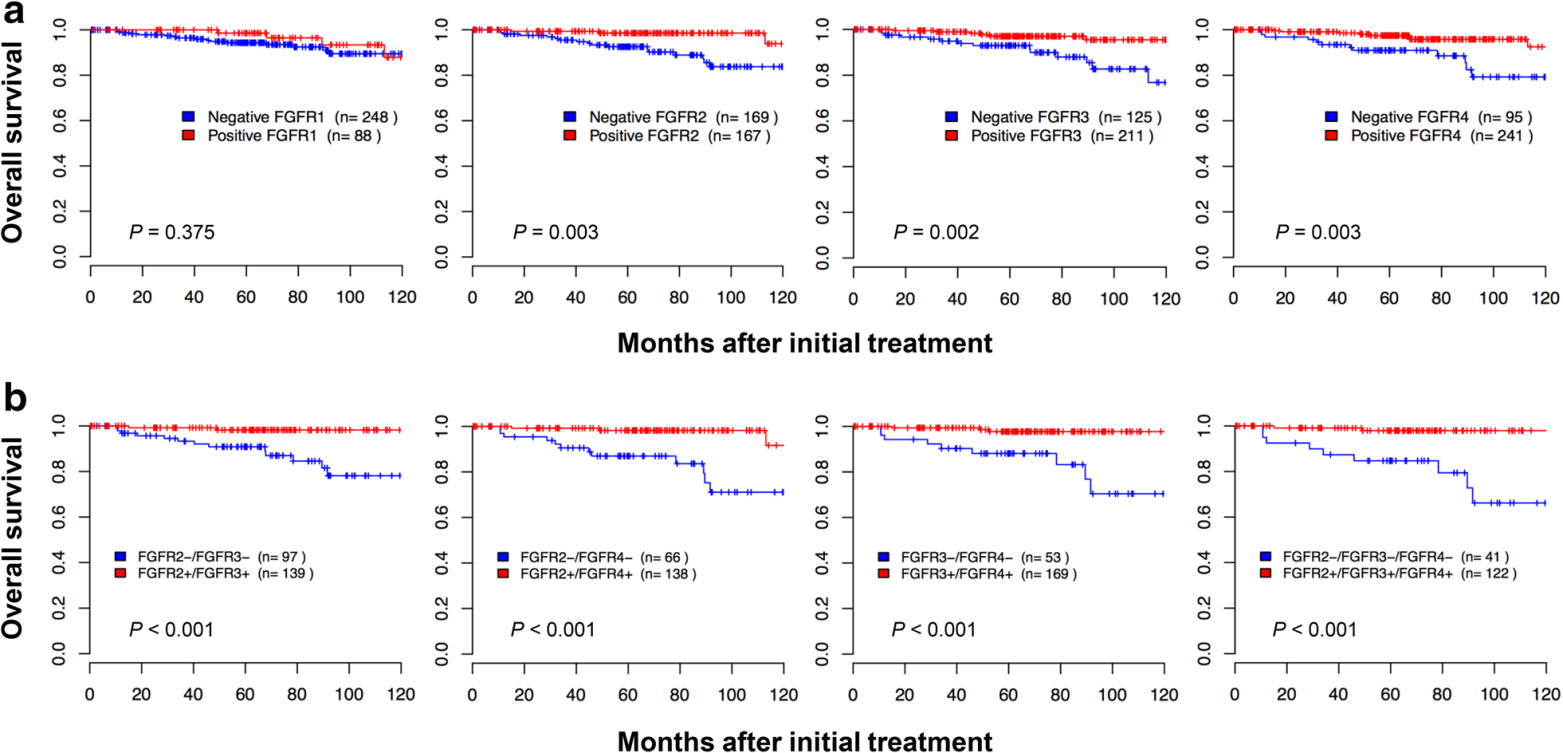
Kaplan–Meier survival curves for overall survival according to FGFR1, FGFR2, FGFR3, and FGFR4. **a** Cervical cancer patients with high FGFR2, FGFR3, and FGFR4 expression had longer overall survival (*P* = 0.003, *P* = 0.002, and *P* = 0.003, respectively) than those with low expression. **b** The combination of FGFR2, FGFR3, and FGFR4 was found to enhance prognostic accuracy for cervical cancer. The patients with FGFR2+/FGFR3+/FGFR4+ expression had significantly longer overall survival (*P* < 0.001) than those with FGFR2-/FGFR3-/FGFR4- expression. *P* values were obtained from log-rank tests

With a median follow-up period of 66 months (range 1–143), five-year disease-free survival and overall survival rates for the whole group were 87 % (95 % CI 83–91) and 96 % (95 % CI 93–98), respectively. A high expression of FGFR2, FGFR3, and FGFR4 was significantly associated with favorable disease-free survival (*P* = 0.006, *P* = 0.035, and *P* = 0.001, respectively) and overall survival (*P* = 0.003, *P* = 0.002, and *P* = 0.003, respectively) (Additional file 2: Figures S3A, S4A). The 5 year disease-free survival was 92, 90, and 91 % for patients with a high expression of FGFR2, FGFR3, and FGFR4, respectively, compared with 81, 82, and 76 % for patients with a low expression. Similarly, the 5 year overall survival was 99, 97, and 97 % for patients with positive FGFR2, FGFR3, and FGFR4 expression respectively, compared with 93, 93, and 91 % for patients with a negative expression (Fig. 4). Furthermore, patients with a combination of high expression of FGFR2 and FGFR3, FGFR2 and FGFR4, or FGFR3 and FGFR4 had significantly more favorable disease-free survival (*P* = 0.004, *P* < 0.001, *P* < 0.001, respectively) and overall survival (all *P* < 0.001) (Fig. 4; Additional file 2: Figures S3B, S4B). Additionally, the coexpression of all three FGFRs was significantly associated with favorable disease-free survival and overall survival (both *P* < 0.001) compared with the single expression of FGFR.

Using the Cox proportional hazards model, the expressions of FGFR2 and FGFR4 remained an independent prognostic factor for disease-free survival [hazard ratio = 0.51 (95 % CI 0.27–0.97), *P* = 0.040; hazard ratio = 0.51 (95 % CI 0.28–0.91), *P* = 0.020, respectively) (Table 3). With respect to overall survival, the expressions of FGFR2, FGFR3, and FGFR4 remained an independent factor [hazard ratio = 0.24 (95 % CI 0.07–0.83), *P* = 0.020; hazard ratio = 0.29 (95 % CI 0.11–0.78), *P* = 0.010; hazard ratio = 0.33 (95 % CI 0.13–0.83), *P* = 0.020, respectively). All the combination markers with FGFR2, FGFR3, and FGFR4 showed independent factors for disease-free survival and overall survival (Additional file 1: Table S3; Table 3). Notably, the prognostic value of FGFR expression was not influenced by cell type.

**Table 3:** Multivariate analysis of the association between prognostic variables and survival in cervical cancer patients

Variables	Disease-free survival	Overall survival
	DFS HR [95 % CI], *P* value	OS HR [95 % CI], *P* value
FIGO stage (IB2/IIB vs. IB1/IIA)	1.72 [0.86–3.47], 0.090	2.00 [0.70–5.73], 0.190
Cell type (AD vs. SCC)	3.80 [2.10–6.86], <0.001	5.89 [2.37–14.65], <0.001
LN metastasis	4.15 [2.20–7.83], <0.001	2.89 [1.12–7.49], 0.030
Tumor size (>4 cm)	0.96 [0.49–1.88], 0.910	0.90 [0.32–2.54], 0.840
PM involvement	1.31 [0.58–2.94], 0.520	1.72 [0.52–5.69], 0.380
FGFR1+	0.52 [0.25–1.11], 0.090	0.48 [0.16–1.50], 0.210
FGFR2+	0.51 [0.27–0.97], 0.040	0.24 [0.07–0.83], 0.020
FGFR3+	0.56 [0.30–1.03], 0.060	0.29 [0.11–0.78], 0.010
FGFR4+	0.51 [0.28–0.91], 0.020	0.33 [0.13–0.83], 0.020
FGFR2+/FGFR3+	0.37 [0.17–0.82], 0.010	0.13 [0.03–0.60], 0.010
FGFR2+/FGFR4+	0.30 [0.13–0.68], 0.004	0.17 [0.05–0.63], 0.008
FGFR3+/FGFR4+	0.33 [0.15–0.69], 0.004	0.11 [0.03–0.44], 0.002
FGFR2+/FGFR3+/FGFR4+	0.24 [1.10–0.60], 0.002	0.08 [0.02–0.40], 0.002

*DFS* disease-free survival; *HR* hazard ratio; *OS* overall survival; *CI* confidence interval; *FIGO* International Federation of Gynecology and Obstetrics; *AD* adenocarcinoma; *SCC* squamous cell carcinoma; *LN* lymph node; *PM* parametrium

Based on the clinical significance of FGFRs protein expressions, the clinical implications of FGFR1, FGFR2, FGFR3, and FGFR4 mRNA expressional levels were assessed. Notably, there was a similar finding between transcription and translation expression levels in cervical cancer. FGFR2 had a significantly favorable prognostic significance at the mRNA level from the GSE44001 dataset (Additional file 2: Figure S5). The RNA-seq dataset of TCGA cervix also showed a favorable prognostic significance of FGFR2 and FGFR3 (Additional file 2: Figure S6).

## Discussion

In the present study, we investigated the prognostic significance of FGFR1, FGFR2, FGFR3, and FGFR4 expression in a large cohort of cervical cancer patients. Here, we identified that the FGFRs were differentially expressed according to cell types of cervical cancer. Furthermore, we demonstrate for the first time that elevated expression of FGFR2, FGFR3, or FGFR4 predict favorable survival in cervical cancer patients. These results suggest that FGFR protein analysis can be a prerequisite in the diagnostic procedure of cervical cancer, and may guide the patients’ therapy. In addition, quantitative digital image analysis used in this study shows potential for more objective results with possible better prognostication. We tested methods of combining the FGFRs expression to predict outcome, as has been demonstrated in the combination of different targets within pathways [33, 34], however were unable to demonstrate a relationship of co-expression that is predictive of outcome.

Published prognostic significances of FGFRs are controversial as there are studies reporting higher recurrence rates with positive FGFR expression, and others finding significantly lower recurrence rates in FGFR expressing tumors [7, 10, 17, 35–37]. FGFR2 has been found to be highly expressed in colorectal cancer and correlated with tumor growth, invasion, and angiogenesis, and stronger FGFR2 expression has been observed in the invasive front of colorectal cancer cells [35]. In addition, there was positive correlation between advanced tumor stage and a high expression of FGFR2 in rectal cancers [36]. On the obverse, down-regulation of FGFR2 in bladder cancers was associated with an adverse prognosis [7]. And, in FGFR2 amplified breast cancer cell lines, constitutive signaling appeared to confer a survival advantage over non amplified cell lines [38]. FGFR3 have also been discussed as a potential prognostic markers in several cancers, however data remain controversial [39–41]. Mutations of the FGFR3 gene are one of the most frequent genetic alterations in bladder cancer and have been shown to be associated with tumors with a favorable prognosis [42–44]. With respect to protein expression, correlation of FGFR3 overexpression with low-grade and low-stage bladder cancers has been reported [6, 7, 37]. Conversely, there are studies reporting an association between the overexpression of the FGFR3 protein and poor survival in breast cancer [45]. There are few studies examining the prognostic significance of FGFR4. In breast cancer, wild type FGFR4 has been proposed to be an important tumor suppressor via the regulation of genes controlling invasion like matrix metalloproteinase 1, suggesting loss of wild type FGFR4 would adversely influence disease progression [46]. Our previous report showed over expression of FGFR4 in rhabdomyosarcoma, and furthermore, activating mutation of FGFR4 can promote metastasis in human rhabdomyosarcomas [19].

These contradictory results may be attributed in part to the cell type specificity. For instance, IIIb and IIIc isoforms of FGFR1 and FGFR2 are expressed in epithelial and mesenchymal cells, respectively [47]. In addition, the cell type specificity can change when FGFRs are associated with cancer. Secondly, the prognostic significance can be different according to mechanism and nature of what is measured. Significance of protein expression can differ from other aberration such as mutation, amplification, or translocations frequently seen in the FGFR pathway. Our present results support the idea that FGFR protein expression is a strong indicator concerning prognosis and survival.

HPV is a well-known etiologic agent in cervical cancer, and persistent infection leads to a genomic instability and local immune suppression, which can lead to both the accumulation of genomic alterations in the host cell, as well as to the integration of the viral genome into the host genome [48]. Recently, genome-wide studies have described the genomic and epigenomic alterations of HPV-associated cancers, which highlighted multiple potential biomarkers and therapeutic targets [49–53]. In addition to recurrent integrations in RAD51B, NR4A2, and TP63, additional genomic alterations were found in receptor tyrosine kinases, primarily FGFR2 and FGFR3, in HPV-positive head and neck squamous cell carcinoma [49]. FGFR2 and FGFR3 mutations have been identified among 17.6 % of HPV-positive tumors, and both mutations have been described in several cancer types, and are sensitive to FGFR inhibitors [54, 55]. Although there is the heterogeneity of HPV-related tumors at different anatomical sites, alteration of FGFR2 and FGFR3 could also be worthwhile to study in cervical cancers.

In the present study, we used quantitative digital image analysis for immunohistochemistry scoring. The advantage of this analysis is, once the analysis protocol has been defined for a given application, large volumes of image data can be processed with minimal user-interaction allowing a highly standardized output on a continuous scale. The prognostic significance of FGFR expression seen in this study, support the usefulness of the image analysis. As quantitative digital image analysis becomes more established, the acknowledgement of results on a continuous scale is likely to become widely accepted and beneficial for improved data mining.

## Conclusions

In conclusion, the present study investigated the immunohistochemical expression of FGFR1, FGFR2, FGFR3, and FGFR4 in a large number of cervical cancer patients. FGFR2, FGFR3, and FGFR4 expression were elevated in squamous cell carcinoma, and a high expression of FGFR1, FGFR2, FGFR3, and FGFR4 were associated with a less aggressive phenotype. Furthermore, a high expression of FGFR2, FGFR3, and FGFR4 showed longer disease-free survival and overall survival. Cox regression analysis confirmed that FGFR2, FGFR3, and FGFR4 expression was an important prognostic indicator in cervical cancer. This information could have clinical value in identifying cervical cancer patients who are at low risk of progression.

## Additional files

10.1186/s12967-016-0874-0 **Table S1.** Antibodies used for immunohistochemistry. **Table S2.** Association between clinicopathological characteristics and two groups defined by cluster analysis. **Table S3.** Univariate analysis of the association between prognostic variables and survival in cervical cancer patients.
10.1186/s12967-016-0874-0 **Figure S1.** Digital image analysis of the nucleus and cytoplasm staining. With the original image (A), nucleus (B) and cytoplasm (C) are classified, and the mean intensity for each fields are presented in a histogram format (D) which enables grouping of the cells by intensity (0, 1+, 2+, and 3+). The final histoscore was calculated by multiplying the intensity and percentage of staining resulting in a range of 0 to 300. Test statistics indicate the discriminative power in the univariate cox model for disease-free survival (R package: survMisc) (E). Nuclear and cytoplasmic area highlighted in green and red, respectively. Dashed vertical lines indicate the chosen cut-off values (FGFR1 = 122, FGFR2 = 58, FGFR3 = 57, and FRGR4 = 79). **Figure S2.** Plots to find the best cut-off values. The p-value of the predicted log-rank test of Kaplan-Meier results was plotted against the normalized staining values. **Figure S3.** Hierarchical clustering analysis for immunohistochemical expression of FGFR1, FGFR2, FGFR3, and FGFR4. (A) Two groups (Category 1 and 2) are defined. Category 1 (n = 116) consists exclusively of high FGFR2 and FGFR3 expression. In contrast, category 2 (n = 220) consists exclusively of low FGFR1, FGFR2, and FGFR3 expression. (B) The patients with Category 1 had significantly longer overall survival (P = 0.024) than those with Category 2. P-values were obtained from log-rank tests. **Figure S4.** Kaplan-Meier survival curves for disease free survival according to FGFR1, FGFR2, FGFR3, and FGFR4. (A) Cervical cancer patients with high FGFR2, FGFR3, and FGFR4 expression had longer disease-free survival (P = 0.006, P = 0.035, and P = 0.001, respectively) than those with low expression. (B) The combination of FGFR2, FGFR3, and FGFR4 was found to enhance prognostic accuracy for cervical cancer. The patients with FGFR2+/FGFR3+/FGFR4+ expression had significantly longer disease-free survival (P < 0.001) than those with FGFR2−/FGFR3−/FGFR4− expression. P-values were obtained from log-rank tests. **Figure S5.** Kaplan-Meier survival curves according to mRNA expression of FGFRs. mRNA expression value was dichotomized according to quartile values (lower than 25 percentile vs. higher than 75 percentile). Data are available at http://www.ncbi.nlm.nih.gov/geo/query/acc.cgi?acc=GSE44001. **Figure S6.** Kaplan-Meier survival curves according to RNA-seq data from TCGA cervix (version: 2015-02-24). mRNA expression value was dichotomized according to quartile values (lower than 25 percentile vs. higher than 75 percentile).

## Abbreviations

FGFR: fibroblast growth factor receptor
FIGO: International Federation of Gynecology and Obstetrics
FGF: fibroblast growth factor
TMA: tissue microarray
GEO: gene expression omnibus
TCGA: The Cancer Genome Atlas
SCC: squamous cell carcinoma
Ag: antigen
AD: adenocarcinoma
ASC: adenosquamous cell carcinoma
LVSI: lymphovascular space invasion
LN: lymph node
PM: parametrium
OP: operation
RT: radiotherapy
CCRT: concurrent chemoradiotherapy
CI: confidence interval
