# Supplementary material for: Expression of fibroblast growth factor receptor family members is associated with prognosis in early stage cervical cancer patients
Source: J Transl Med. 2016 May 6;14:124. doi: 10.1186/s12967-016-0874-0 (PMC4859953; doi:10.1186/s12967-016-0874-0)
Supplement: Supplementary file 1 — 10.1186/s12967-016-0874-0 Table S1. Antibodies used for immunohistochemistry. Table S2. Association between clinicopathological characteristics and two groups defined by cluster analysis. Table S3. Univariate analysis of the association between prognostic variables and survival in cervical cancer patients. [file 12967_2016_874_MOESM1_ESM.pdf]

Supplementary Table 1 Antibodies used for immunohistochemistry

Protein	Cat. No.	Clone	Vendor	Dilution	Incubation
FGFR1	#ab10646	Polyclonal rabbit	Abcam, Cambridge, UK	1:2000	RT, 1 hr
FGFR2	#ab10648	Polyclonal rabbit	Abcam, Cambridge, UK	1:2000	RT, 1 hr
FGFR3	#sc-13121	Monoclonal mouse, B-9	Santa Cruz, CA, USA	1:50	4ºC, Overnight
FGFR4	#sc-124	Polyclonal rabbit, C-16	Santa Cruz, CA, USA	1:200	RT, 2 hr

RT, room temperature

Supplementary Table 2 Association between clinicopathological characteristics and two groups defined by cluster analysis

	Category 1 (%)	Category 2 (%)	*P* value*
**Age**			
< 50 yrs	63 (31)	140 (69)	0.100
> 50 yrs	53 (40)	80 (60)	
**FIGO Stage**			
IB1/IIA	101 (35)	190 (65)	0.860
IB2/IIB	15 (33)	30 (67)	
**Cell type**			
SCC	102 (40)	154 (60)	<0.001
AD/ASC	14 (18)	66 (82)	
**Tumor size**			
≤ 4 cm	96 (38)	160 (62)	0.040
> 4 cm	20 (25)	60 (75)	
**LVSI**			
Negative	72 (36)	130 (64)	0.530
Positive	43 (32)	90 (68)	
**Depth of invasion**			
< 50%	40 (37)	68 (63)	0.500
> 50%	76 (33)	152 (67)	
**LN metastasis**			
Negative	97 (38)	159 (62)	0.020
Positive	19 (24)	61 (76)	
**PM involvement**			
Negative	108 (35)	197 (65)	0.280
Positive	8 (26)	23 (74)	
**Primary Treatment**			
OP only	72 (42)	99 (58)	0.022
OP + RT	20 (29)	50 (71)	
OP + CCRT	22 (24)	68 (76)	
Neoadjuvant	2 (40)	3 (60)	

*Chi-square test

FIGO, International Federation of Gynecology and Obstetrics; SCC, squamous cell carcinoma; Ag, antigen; AD, adenocarcinoma; ASC, adenosquamous cell carcinoma; LVSI, lymphovascular space invasion; LN, lymph node; PM, parametrium; OP, operation; RT, radiotherapy; CCRT, concurrent chemoradiotherapy

Supplementary Table 3 Univariate analysis of the association between prognostic variables and survival in cervical cancer patients

Variables	Disease-free survival	Overall survival
	**HR [95% CI], *P* value**	**HR [95% CI], *P* value**
**FIGO stage (IB2/IIB vs. IB1/IIA)**	2.44 [1.24 – 4.82], 0.01	2.49 [0.90 – 6.87], 0.08
**Cell type (AD vs. SCC)**	2.88 [1.62 – 5.14], <0.001	4.52 [1.85 – 11.06], <0.001
**LN metastasis**	4.13 [2.31 – 7.38], <0.001	2.93 [1.22 – 7.06], 0.02
**Tumor size (> 4 cm)**	1.70 [0.92 – 3.15], 0.09	1.48 [0.57 – 3.86], 0.42
**PM involvement**	2.24 [1.05 – 4.81], 0.04	2.83 [0.94 – 8.48], 0.06
**FGFR1 +**	0.63 [0.30 – 1.30], 0.21	0.61 [0.20 – 1.83], 0.38
**FGFR2 +**	0.43 [0.23 – 0.80], 0.01	0.19 [0.05 – 0.63], 0.01
**FGFR3 +**	0.54 [0.30 – 0.97], 0.04	0.25 [0.10 – 0.65], 0.005
**FGFR4 +**	0.39 [0.22 – 0.70], 0.002	0.28 [0.11 – 0.68], 0.005
**FGFR2 +/FGFR3 +**	0.34 [0.16 – 0.73], 0.006	0.11 [0.02 – 0.49], 0.004
**FGFR2 +/FGFR4 +**	0.23 [0.10 – 0.51], <0.001	0.13 [0.04 – 0.46], 0.002
**FGFR3 +/FGFR4 +**	0.28 [0.14 – 0.59], <0.001	0.11 [0.03 – 0.38], <0.001
**FGFR2+/FGFR3+/FGFR4+**	0.22 [0.09 – 0.55], 0.001	0.08 [0.02 – 0.38], 0.001

CI, confidence interval; FIGO, International Federation of Gynecology and Obstetrics; AD, adenocarcinoma; SCC, squamous cell carcinoma; LN, lymph node; PM, parametrium
