# Supplementary figures and images for: Expression of fibroblast growth factor receptor family members is associated with prognosis in early stage cervical cancer patients
Source: J Transl Med. 2016 May 6;14:124. doi: 10.1186/s12967-016-0874-0 (PMC4859953; doi:10.1186/s12967-016-0874-0)

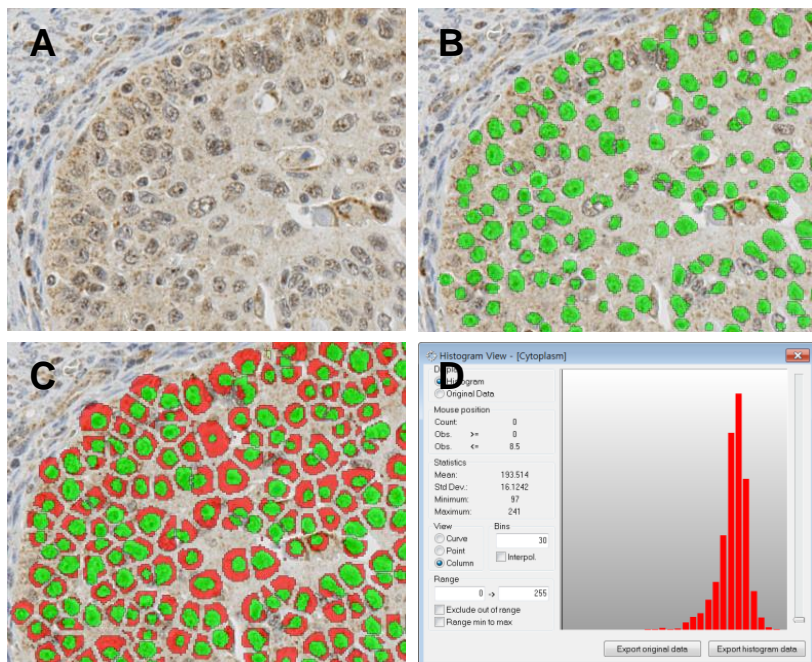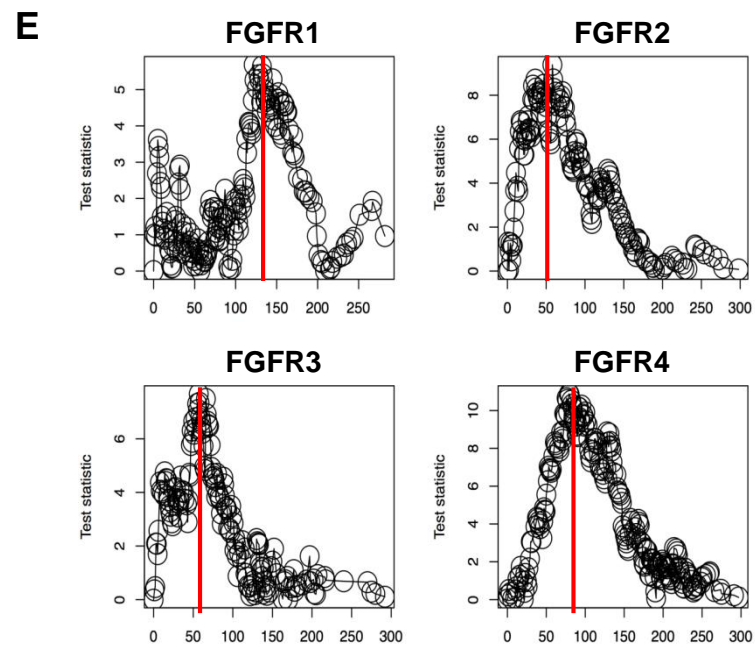

Figure S1

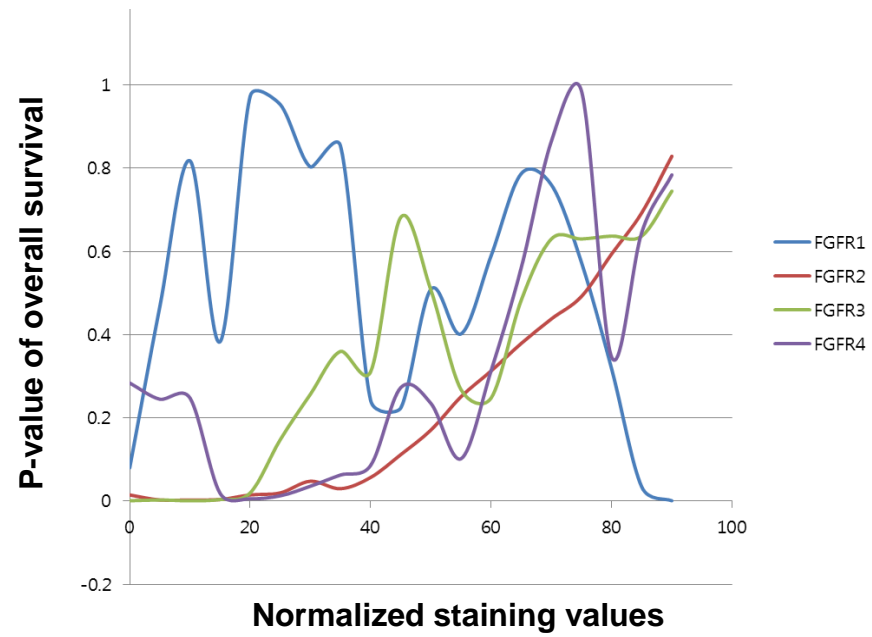

**Figure S2**

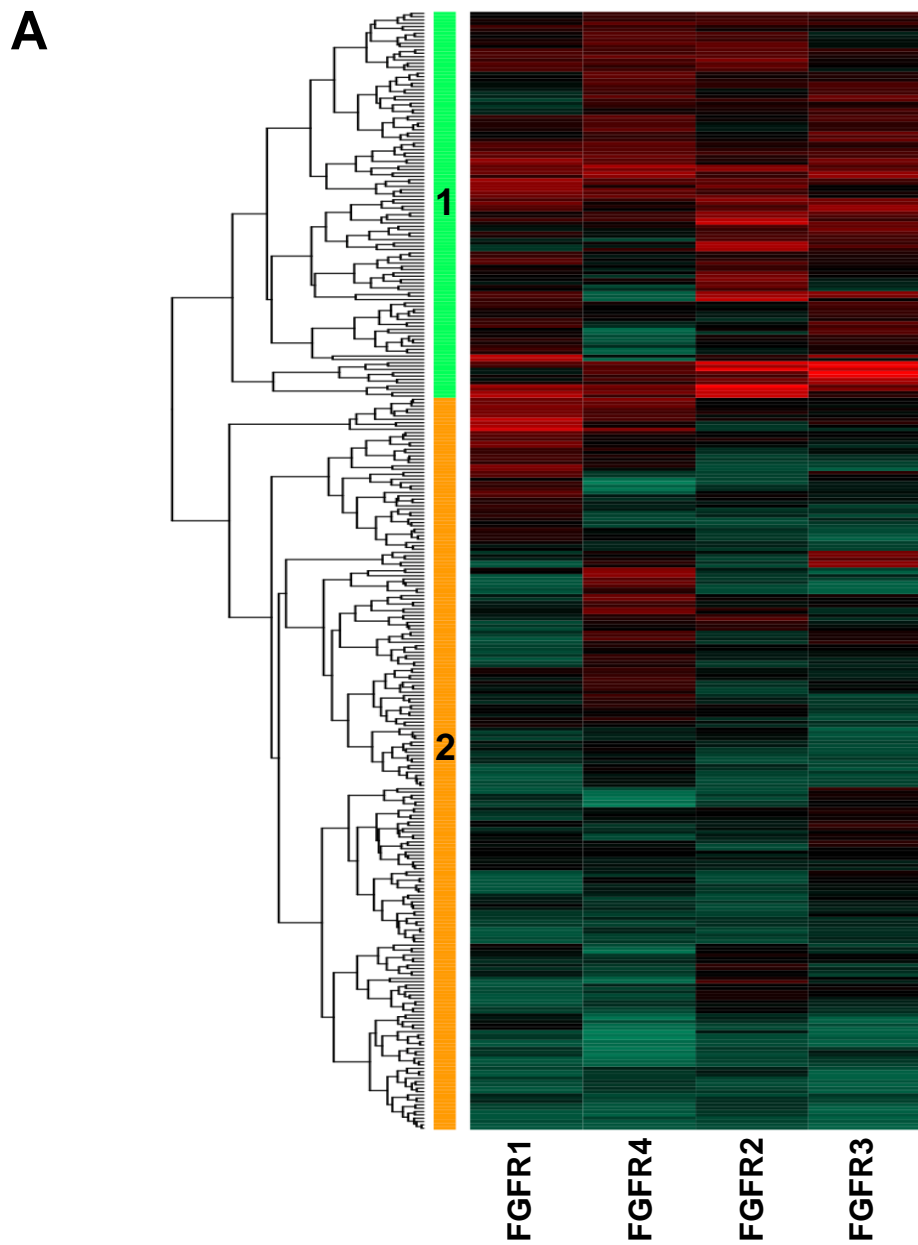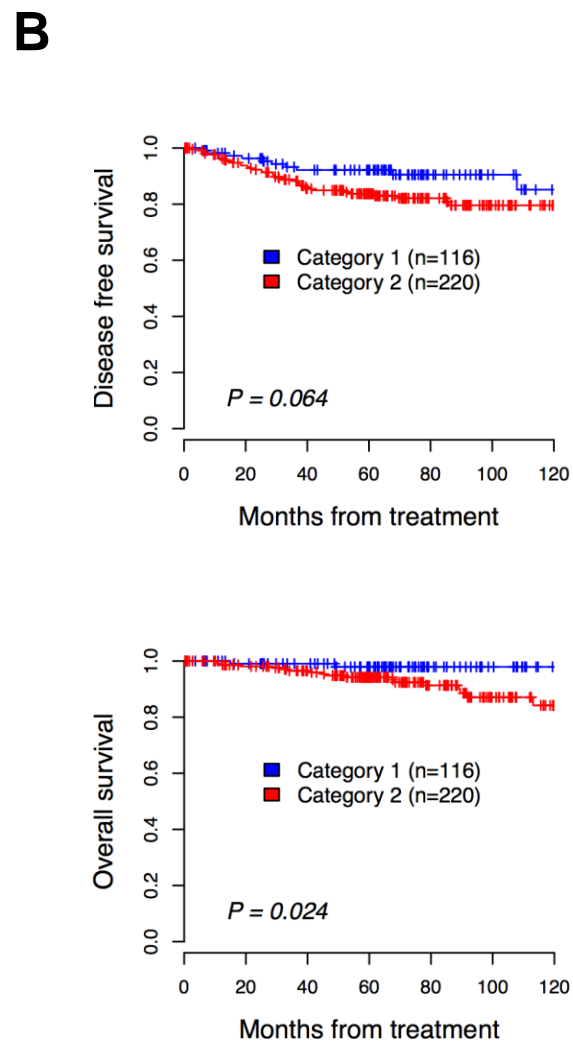

Figure S3

**A**

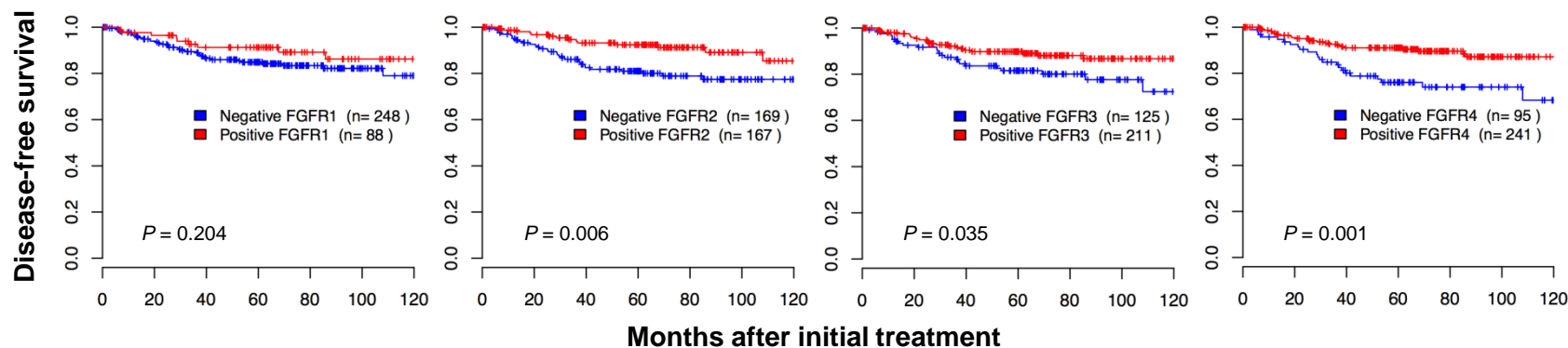

**B**

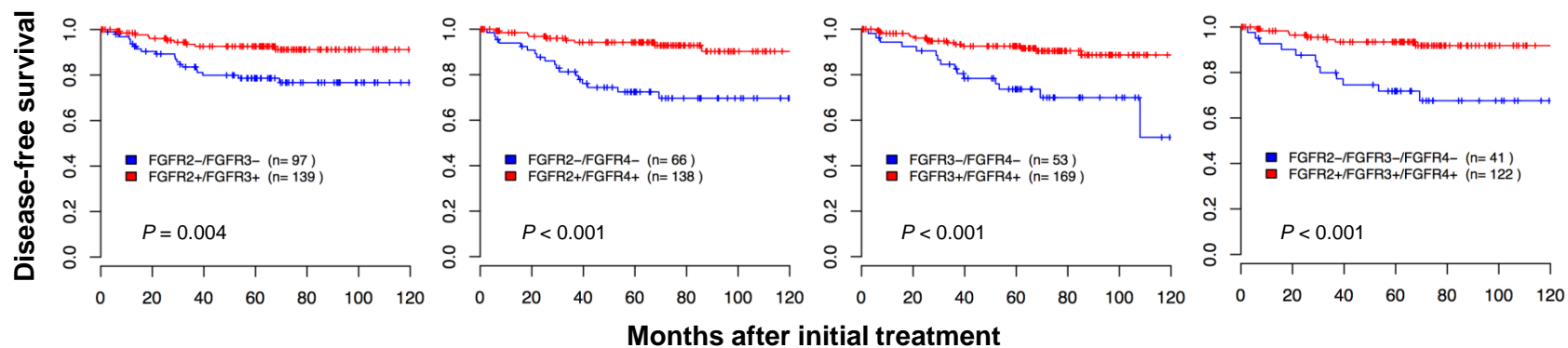

**Figure S4**

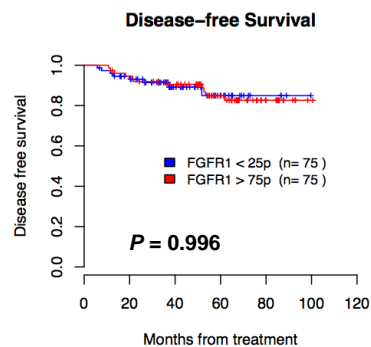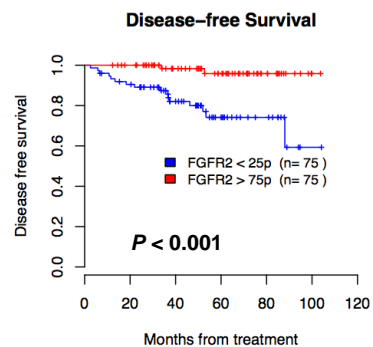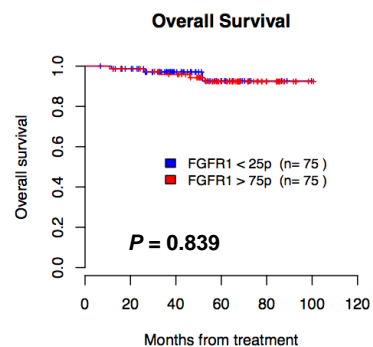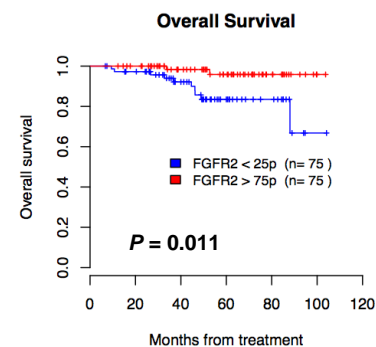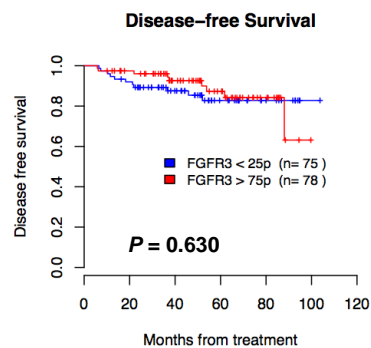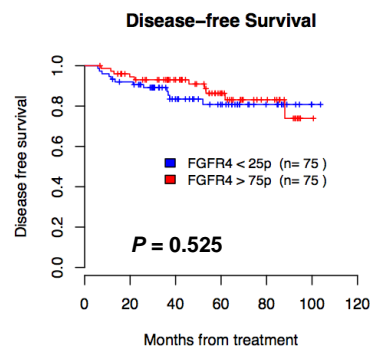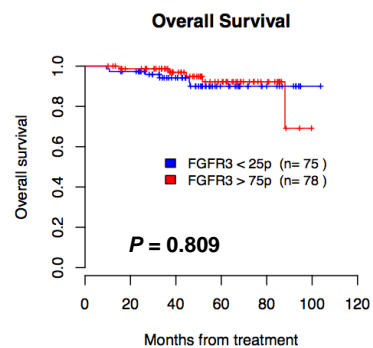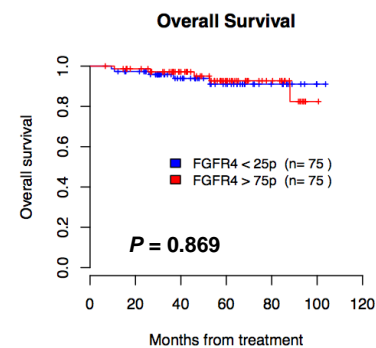

**Figure S5**

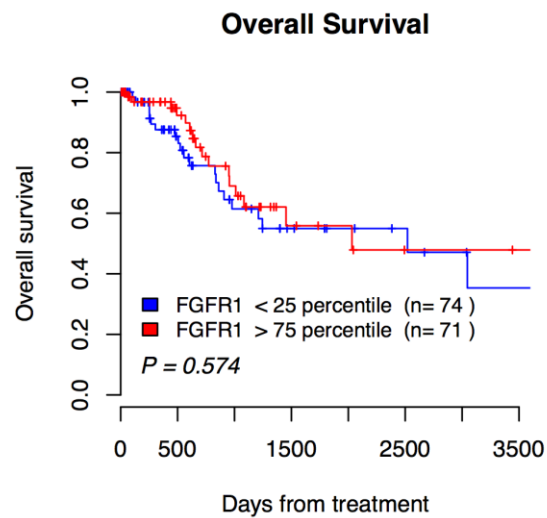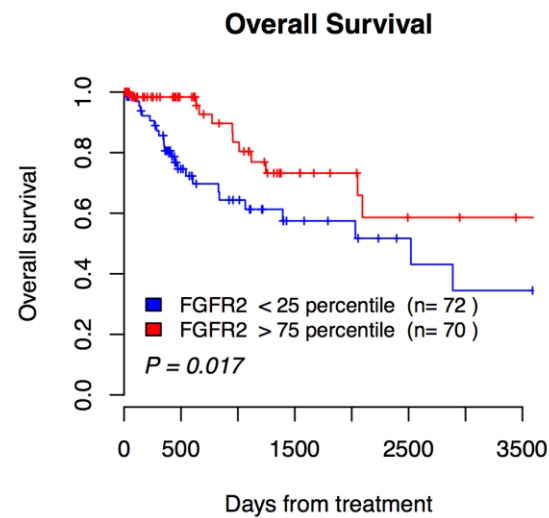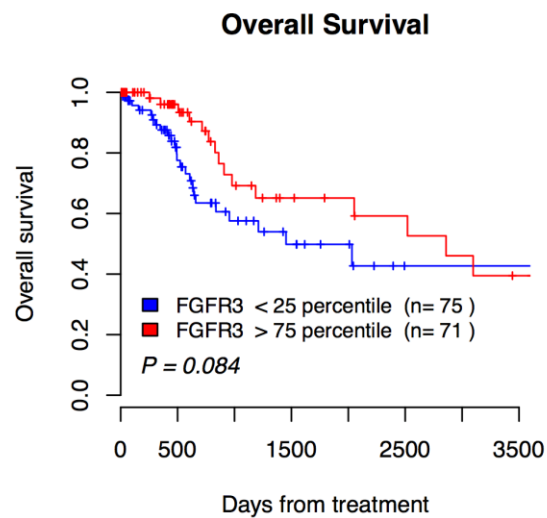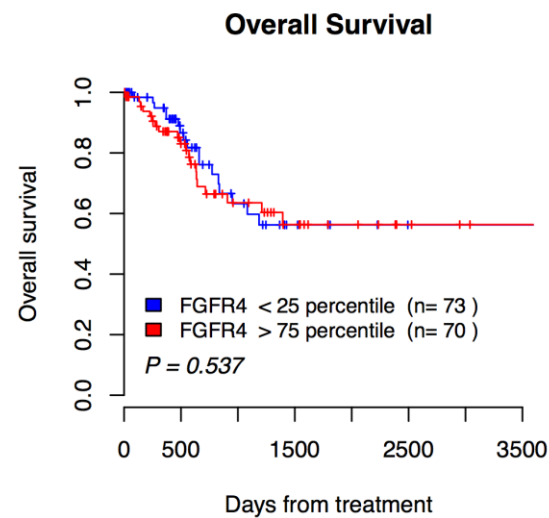

**Figure S6**

Supplement: Supplementary file 2 — 10.1186/s12967-016-0874-0 Figure S1. Digital image analysis of the nucleus and cytoplasm staining. With the original image (A), nucleus (B) and cytoplasm (C) are classified, and the mean intensity for each fields are presented in a histogram format (D) which enables grouping of the cells by intensity (0, 1+, 2+, and 3+). The final histoscore was calculated by multiplying the intensity and percentage of staining resulting in a range of 0 to 300. Test statistics indicate the discriminative power in the univariate cox model for disease-free survival (R package: survMisc) (E). Nuclear and cytoplasmic area highlighted in green and red, respectively. Dashed vertical lines indicate the chosen cut-off values (FGFR1 = 122, FGFR2 = 58, FGFR3 = 57, and FRGR4 = 79). Figure S2. Plots to find the best cut-off values. The p-value of the predicted log-rank test of Kaplan-Meier results was plotted against the normalized staining values. Figure S3. Hierarchical clustering analysis for immunohistochemical expression of FGFR1, FGFR2, FGFR3, and FGFR4. (A) Two groups (Category 1 and 2) are defined. Category 1 (n = 116) consists exclusively of high FGFR2 and FGFR3 expression. In contrast, category 2 (n = 220) consists exclusively of low FGFR1, FGFR2, and FGFR3 expression. (B) The patients with Category 1 had significantly longer overall survival (P = 0.024) than those with Category 2. P-values were obtained from log-rank tests. Figure S4. Kaplan-Meier survival curves for disease free survival according to FGFR1, FGFR2, FGFR3, and FGFR4. (A) Cervical cancer patients with high FGFR2, FGFR3, and FGFR4 expression had longer disease-free survival (P = 0.006, P = 0.035, and P = 0.001, respectively) than those with low expression. (B) The combination of FGFR2, FGFR3, and FGFR4 was found to enhance prognostic accuracy for cervical cancer. The patients with FGFR2+/FGFR3+/FGFR4+ expression had significantly longer disease-free survival (P < 0.001) than those with FGFR2−/FGFR3−/FGFR4− expression. P [file 12967_2016_874_MOESM2_ESM.pdf]
